# Valuable insights into general practice staff's experiences and perspectives on AI-assisted diabetic retinopathy screening—An interview study

**DOI:** 10.3389/fmed.2025.1565532

**Published:** 2025-03-11

**Authors:** Malene Krogh, Malene Hentze, Morten Sig Ager Jensen, Martin Bach Jensen, Marie Germund Nielsen, Henrik Vorum, Jette Kolding Kristensen

**Affiliations:** ^1^Center for General Practice at Aalborg University, Aalborg, Denmark; ^2^Department of Otorhinolaryngology, Head and Neck and Audiology, Aalborg University Hospital, Aalborg, Denmark; ^3^The Clinical Nursing Research Unit, Aalborg University Hospital, Aalborg, Denmark; ^4^Department of Health Science and Technology, Aalborg University, Aalborg, Denmark; ^5^Department of Ophthalmology, Aalborg University Hospital, Aalborg, Denmark

**Keywords:** diabetic retinopathy screening, artificial intelligence, machine learning, primary care, stakeholder perspectives, qualitative research

## Abstract

**Aim:**

This study explores the hands-on experiences and perspectives of general practice staff regarding the feasibility of conducting artificial intelligence-assisted (AI-assisted) diabetic retinopathy screenings (DRS) in general practice settings.

**Method:**

The screenings were tested in 12 general practices in the North Denmark Region and were conducted as part of daily care routines over ~4 weeks. Subsequently, 21 staff members involved in the DRS were interviewed.

**Results:**

Thematic analysis generated four main themes: (1) Experiences with DRS in daily practice, (2) Effective DRS implementation in general practice in the future, (3) Trust and approval of AI-assisted DRS in general practice, and (4) Implications of DRS in general practice. The findings suggest that general practice staff recognise the potential for AI-assisted DRS to be integrated into their clinical workflows. However, they also emphasise the importance of addressing both practical and systemic factors to ensure successful implementation of DRS within the general practice setting.

**Conclusion:**

Focusing on the practical experiences and perspectives of general practice staff, this study lays the groundwork for future research aimed at optimising the implementation of AI-assisted DRS in general practice settings, while recognising that the insights gained may also inform broader primary care contexts.

## 1 Introduction

The use of artificial intelligence (AI) in healthcare has gained significant attention, with primary care expected to play a key role in unlocking its full potential by implementing AI at the patient's first point of contact (1, 2).

One of the leading areas of AI development is in the screening and diagnosis of diabetic retinopathy (DR) (3), enabling task delegation to primary care staff (4, 5). This shift offers advantages, as AI-assisted screenings can be performed closer to patients, enhancing follow-up care participation (6, 7). Research on DR screening (DRS) in primary care has primarily focused on algorithm accuracy (8–11). However, accuracy alone does not address the practical integration of DRS into clinical practice. A key challenge understands how AI-assisted DRS can be integrated into existing workflow as well as staff's perspectives on its implementation.

There is an increasing number of studies investigating healthcare staff's perspectives on factors influencing implementation of DRS in primary care (5, 12–17). Among the various perspectives, key insights include the potential value of screenings for enhancing eye health care (5), the need for adequate training (15, 16), importance of financial considerations (13–15), ease of use (5, 15), challenges during screening (5, 12), and the trustworthiness of AI-assisted DRS (4, 15). However, perspectives of screening possibility often occur before staffs have had the opportunity to use DRS in the intended clinical setting (13, 14).

Previous experiences with the implementation of DRS in primary care settings have demonstrated that even with a carefully designed protocol developed in collaboration with stakeholders, unforeseen challenges may still emerge during the execution phase (12). In a study by Beede et al. (12), the implementation of DRS in a Thai clinical setting faced unexpected challenges, including inadequate image quality due to external environmental factors, long waiting times from poor patient screening organisation, and technical issues that forced staff to deviate from the planned protocol to prioritise patient care. These findings underscore the importance of real-world clinical testing to assess feasibility of DRS.

Research indicates that AI tools integrated naturally into existing workflows are more likely to be accepted by clinicians (18). It is therefore essential to involve stakeholders in trial planning and evaluation while learning from previous studies. Existing literature on DRS in primary care provides insights into staff perspectives on factors affecting successful implementation (5, 12–17, 19). However, directly applying methods and findings across healthcare systems is challenging due to variations between countries.

In Denmark, the management and primary care of patients with type 2 diabetes (T2D) are primarily conducted in general practice, while DRS are typically performed in private ophthalmologists' practices. There is a shortage of ophthalmologists and long waiting lists for consultations (20). Approximately 82% of Danish patients with T2D do not have DR (21). Given the high demands on ophthalmology services, alternative screening approaches would be helpful, particularly considering emerging AI-assisted screenings. Patients with T2D frequently visit general practice for ongoing management, so incorporating DRS into a routine T2D visits could reduce the need for ophthalmologist consultations, as only patients with detected DR alterations would require referral to an ophthalmologist.

General practice staff plays a central role in successful implementation of AI-assisted DRS. By providing them with hands-on experience, we can gain valuable insights from staff which are essential for future planning and rollout of DRS in general practice. We performed a feasibility study testing AI-assisted DRS in general practices, aiming to explore staff experiences and perspectives on conducting DRS as part of diabetes care.

## 2 Method

### 2.1 Study design, participants, and setting

In this qualitative, cross-sectional study staff from 12 general practices in the North Denmark Region participated in conducting DRS during T2D diabetes consultations. Staffs were selected by the practices based on their involvement in T2D care, and one or more staff members from each practice participated. No exclusion criteria were applied to staff. Practices were compensated for their time spent on the study.

### 2.2 AI-assisted DRS

AI-Assisted DRS was conducted using a non-mydriatic fundus camera (FundusScope, Rodenstock, Germany) (22) with a field view of 45°. The camera has a 12-megapixel sensor and a LED flash which captures high-resolution images (4,096 × 3,072). The camera is designed to require minimal user involvement, with staff primarily responsible for ensuring the correct positioning of the patient. To initiate the image capture process, staff had to press a START button; thereafter, the camera automatically captured the retinal image while independently managing zoom and flash. To minimise the impact of the flash on the pupil from the first image, staffs were advised to wait 20 s before capturing a photo of the second eye. Dilating eyedrops was not provided on the patients.

Captured images were automatically sent to a cloud-based AI analysis software (RetinaLyze System; RetinaLyze A/S, Hørsholm, Denmark) (23). The software utilises Support Vector Machine Learning (SVML), enabling it to classify the presence or absence of visible retinal changes, and has been available on the market since 2013. The software operates by detecting red lesion in an image and all detected red lesions are marked with a black circle. The number of red lesions determines the screening result. The result of the analysis is displayed as a colour code: green for no DR alterations, yellow for few alterations, red for several alterations (>3) or grey for poor image quality, which required recapturing. An AI-analysis is performed in ~15 s. The software was provided free of charged by RetinaLyze during the testing period.

### 2.3 Procedure

#### 2.3.1 Practices begin patient recruitment

**Figure 1:**
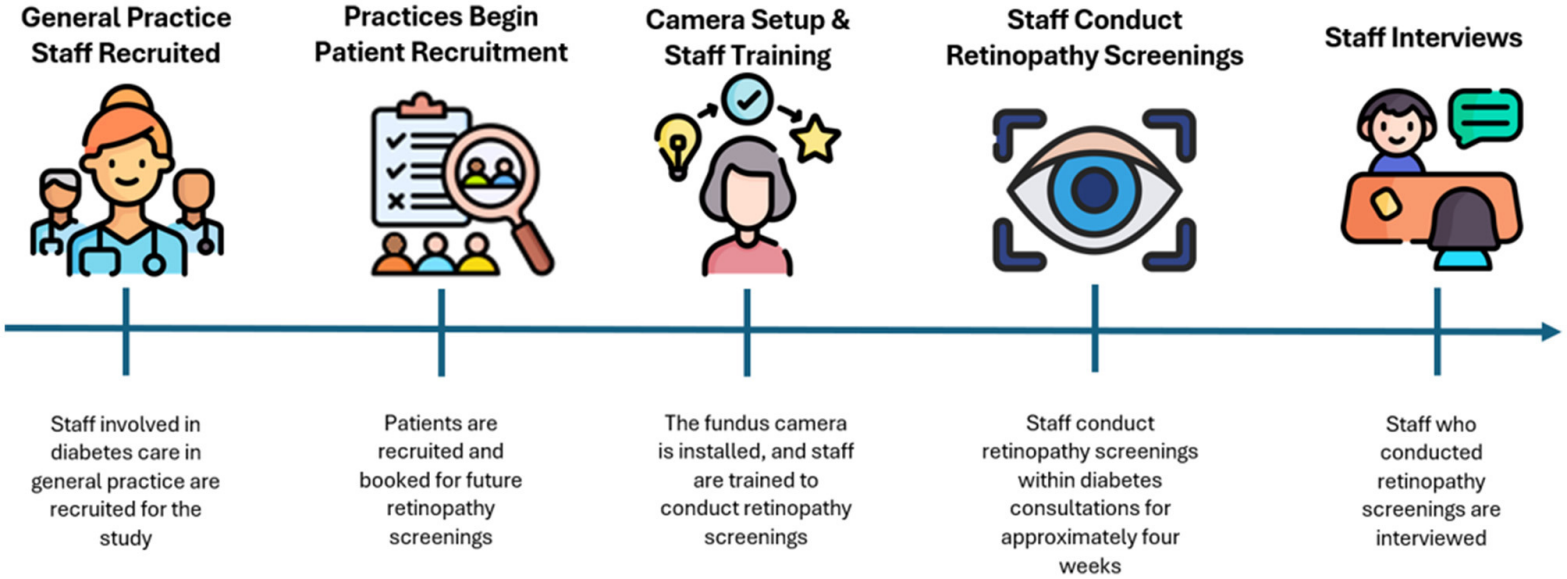
Timeline of events in the study.

Practices were responsible for recruiting patients for DRS and practices were provided with the necessary materials to begin recruitment prior to the testing period (see Figure 1 for an illustration of the timeline of events in the study). A few inclusion criteria had to be met for patients: they needed to have T2D, be able to understand Danish, be 18–70 years of age, and not be blind. Patients were required to participate in a follow-up screening at a collaborating private ophthalmologist practice in the North Denmark Region. Due to recruitment difficulties, patients from the last two recruited practices were not re-screened at the collaborating private ophthalmology practice. A total of 298 patients were recruited, each receiving written and verbal information about the project and providing written informed consent prior to participation.

#### 2.3.2 Camera setup and staff training

Prior to conduction DRS, the camera was setup in the practice. The placement of the camera was determined in collaboration with the staff to ensure optimal positioning, considering room availability and the need to minimise light interference. A large cardboard was used to cover light interference from windows in some practices. When possible, the camera was connected to the practice's internet via Wi-Fi or Ethernet cable, facilitating the AI analysis. In cases where the practice's internet was unavailable, the camera was connected to a mobile hotspot on a tablet (Huawei, MediaPad T3 10). Verbal and written instructions on connecting the camera to the hotspot were provided to staff.

On the same day as camera setup, staff participated in a training session initiated by MK or MH. Staff underwent training in both theoretical and practical aspects of the screening process through instruction videos and hands-on exercises (16). The session covered operating the camera, image quality assessment, uploading images to the AI analysis software, communicating results to patients, and providing guidance on potential solutions to issues that might arise during the screening process. The session was held within the intended screening location within the practice. The training is developed by the author group, and the process has been describes in detail (16).

#### 2.3.3 Staff conduct retinopathy screenings

Each practice had access to the DRS equipment for ~4 weeks. Practices were granted a high degree of autonomy regarding the integration of the screening into their existing routines and were given the flexibility to decide whether to conduct screenings during a separate consultation or as part of an existing appointment. Staffs were instructed to inform patients of the screening results. Each practice was advised to allocate 20 min per screening session, as they were also required to collect supplementary data following the screening (which is not part of this study).

#### 2.3.4 Staff interviews

Semi-structured interviews were conducted by MK or MH with staff in the clinical setting (office or consultation room) after each practice had completed the conduction of DRS (24). The interview aimed to explore staff experiences and perspectives on DRS in general practice. An interview guide was developed by the author group (24) which guided the interviewers during the interviews. While it provided structure, the guide allowed flexibility to explore new insights rather than strictly following a predetermined set of questions. The number of interview participants was dependent on the number of staff in the general practice clinics conducting the screening. The interviews were conducted in Danish and were audio-recorded on a Sony ICD-PX370 Digital Dictaphone. Interviews were performed from April 2022 to December 2023. The interviewers are both early-career researchers without much experience in conducting interviews. To prepare for conducting and analysing interviews, they had reviewed relevant literature and attended qualitative research courses. MH has a medical background, while MK has an educational background in sport science. At the time of the interviews, both interviewers were employed at a research centre for general practice. Neither MK nor MH had a personal relationship with any of the participants.

### 2.4 Data analysis

An inductive thematic analysis was carried out by MK in accordance with analytical principles and guidelines (25). Initially, all interviews were transcribed verbatim in Microsoft Word before being imported into NVivo 14 for analysis. During transcription, preliminary notes were made. Further familiarisation of data was achieved by reading all transcriptions, while still making notes. When familiarised sufficiently with the data, codes and sub-codes were developed by identifying recurring patterns and perspectives relevant to the aims. Themes and sub-themes were generated based on the analysis of codes. The generation of both codes and themes was an iterative process, which required frequent revisions to the transcriptions or codes to enhance the clarity and precision of both codes and themes. Throughout the analytical process, MK engaged in collaborative discussions with MH to ensure quality assurance at each step. MH reviewed five transcription, and several meetings were conducted to examine codes, themes, and the overall context of the findings. Selected quotes were included to demonstrate how the findings are reflected in the data. The transcriptions, codes, and themes were analysed in Danish. The translation of themes and quotes into English was performed during the final write-up of the article by MK, who is fluent in both Danish and English but is not a native English speaker. Care was taken to preserve meaning during the translation process, though some linguistic nuances may have been altered.

### 2.5 Ethical approval

This study was approved by the Committee on Medical Research Ethics, protocol number: 2200781. Staff gave informed consent for participating in the interview.

## 3 Results

The 21 staff members who were interviewed are detailed in Table 1. The average age of the staff was 51 ± 6 years and the number of staff participating in each clinic ranged from 1 to 5. Interviews lasted an average of 36 min, ranging from 22 to 60 min.

**Table 1:** Staff characteristics.

**Staff no**.	**Gender**	**Age**	**Job title**
P1	Male	59	General practitioner
P2	Female	47	Social and health assistant
P3	Female	49	Biomedical laboratory scientist
P4	Female	56	Nurse
P5	Female	49	Nurse
P6	Female	61	Nurse
P7	Female	65	Nurse
P8	Female	37	Nurse
P9	Female	52	Nurse
P10	Female	55	Nurse
P11	Female	51	Nurse
P12	Female	44	Nurse
P13	Female	51	Nurse
P14	Female	44	Nurse
P15	Female	46	Nurse
P16	Female	52	Nurse
P17	Female	48	Nurse
P18	Female	44	Nurse
P19	Female	65	Nurse
P20	Female	48	Nurse
P21	Female	59	Nurse

### 3.1 Themes

Four main themes were generated from interviews with associated sub-themes, as presented in Table 2.

**Table 2:** Overview of generated themes and sub-themes.

**Themes**	**Sub-themes**
Conducting DRS in daily practice	Logistics adaptations
	Quick and easy screening
	Problems lead to action
	Team dynamics in screening
	Reactions and communication on screening results
Effective DRS implementation in general practice in the future	Spatial and ergonomic requirements
	Planning and coordination needs
	Staff desire for more knowledge
	Screening in annual consultation
	Financial conditions
	Defined plan for ophthalmologist involvement
Trust and approval of AI-assisted screening in general practice	Approval of DRS in general practice
	Validity as the foundation for technological trust and implementation
	AI as a screening tool
Implications of DRS in general practice	Increased convenience and attendance
	Avoidance of ophthalmology visits
	Increased responsibilities in general practice

DRS, Diabetic retinopathy screening; AI, Artificial intelligence.

#### 3.1.1 Conducting DRS in daily practice

##### 3.1.1.1 Logistics adaptations

Staff experiences with DRS in daily practice involved several logistical adaptations, including camera positioning, calendar planning and organisation of the screening process. Camera placement varied across practices, depending on available spaces and workflow. In some practices, the camera was placed on staff desks or in undisturbed hallway areas, while others had it placed in unused or shared consultation rooms:

“*We've done everything in our Laboratory 2. On Mondays and Fridays, there are others in there, so I just switch rooms with them, and they sit in my office taking blood samples.”* (P18)

Screenings were scheduled in staff work calendars, with some staff using colour-coded entries to indicate when multiple staff members were involved in screenings, to ensure there were no double bookings of the room and camera.

Screenings were either integrated into upcoming diabetes consultations with additional time allocated or scheduled as separate consultations dedicated solely to DRS. Some practices exclusively conducted separate screenings in block-booked sessions, while others adopted a mixed approach, combining both methods:

“*We started by reviewing our calendar to see patients who were already due for a check-up… Some appointments were moved up to combine the screening with their visit, while others just booked a time directly. On certain days, we scheduled several in a row, making it more efficient.”* (P15)

In all practices, 10–20 min were allocated per patient screening.

##### 3.1.1.2 Quick and easy screening

All staff members reported that the screening procedure was quick and easy to perform, often completed in just a few minutes.

“*Most of them take 2, 3, 4 minutes, so the examination itself is really quick and really easy.” (P10)*

Patient interactions generally went well, and staff noted that guiding patients into position at the camera was easy. The procedure was straightforward to explain to patients, and several staff members observed that many patients were already familiar with the camera.

“*Many of them have already tried it at the ophthalmologist. It's actually the same, right? So, they know when I ask, “Have you tried this before?” “Yeah, I need to sit here.” So, they understood. It didn't take that long, and I could also see that the further we got into it, the quicker the screening went.”* (P2)

##### 3.1.1.3 Problems lead to action

Staff faced patient-specific challenges, such as strabismus, which hindered camera focus, and disturbances from eyelashes or eyelids obstructing the lens. Internet connectivity issues caused delays in analysis or prevented image uploads for some. An often-reported issue was the presence of small pupils. For some staff, this was attributed to lighting conditions in the screening room, and creative solutions were attempted to minimise light interference. In a few cases, capturing images was not possible due to small pupils, while in other instances, the issue was resolved by waiting longer between photos.

“*There was one instance where we actually had to give up, when my colleague called me in, and we had to give up on a patient because it kept saying that the pupils were too small, and no matter what we did, we just couldn't adjust it. I've also experienced this with one other patient, but then we just took a longer break, and we were allowed to take the second picture. Otherwise, I haven't encountered any problems with it.”* (P15)

One staff member encountered technical issues with the camera and the interaction between its components, particularly problems arising from the hotspot connexion.

“*At first, I didn't have control over the hotspot that needed to be connected, and I had to make sure everything was started in the correct order, and then the hotspot was suddenly disconnected again. I couldn't get it to work together… It was really frustrating because the actual image capture was easy, but all the technical aspects just didn't work together.”* (P7)

##### 3.1.1.4 Team dynamics in screening

Staff consulted with each other throughout the process and described having multiple people involved in the screening as an advantage.

“I actually think it worked well that both of us did it, that both of us knew how to do it, both of us were trained... And if we had any issues, we could just help each other out.” (P4)

In a clinic where five diabetes care providers participated, collaboration and mutual support were described; however, it also presented some obstacles in terms of developing a consistent routine.

“*We've talked a bit about how we shouldn't have been five people. It would have been fine with two. But we all thought the project was exciting. But we suddenly got into it quickly, and the next day I had screenings again, and suddenly some people had to start over, like from the beginning, they had to get familiar with the camera and get the routine, all those things. So, the results become different, because it can take longer for everyone to get into it, especially when new people come in and take over.” (*P21)

##### 3.1.1.5 Reactions and communication on screening results

Most staff were confident communicating results to patients; however, a few were more comfortable providing a green result, believing that yellow or red results could cause patient anxiety. Most results were green, while yellow and red results were described as rare or never encountered.

“*I maybe had one yellow, that's it; everyone else had green, and I couldn't say anything other than it was green. I felt comfortable with that… It's not that I didn't trust the result; my concern was more about how the patient might feel if the result was red and they became more anxious.”* (P20)

For yellow and red results, most staff reassured patients by clarifying that the results were part of a preliminary test and not definitive. In practices where follow-up screening had not been pre-arranged, staff recommended that patients with yellow or red results followed-up with their own ophthalmologist.

“*I would recommend it, it was completely natural for me to say, ‘You know what, you need to see the ophthalmologist, this is important.' So, it was a good way to start the conversation, and then to say, ‘This machine is showing something, so it's a good idea that you go see your ophthalmologist.' This happened with the patient who had the red result. He's the only one I had with a red result, and I could really feel, oh, he really needs to see the ophthalmologist.” (P21)*

Staff noted that patients generally did not express concern over their results, were aware of DR alterations and often compared results with previous ophthalmologist results.

Staff generally expressed trust in the accuracy of results. However, they also acknowledged that the screening was part of a study and approached the results with some caution. The reassurance that patients would still be referred to an ophthalmologist for follow-up made staff feel more at ease when receiving a result.

“*I actually think I trusted it— I really do. I thought, ‘Well, that's nice,' when the result came back green. Of course, I'm aware that it's still a project, I know that. So, I sort of trust it… But it's definitely reassuring to know that they'll still go to the ophthalmologist.”* (P13)

Three staff members had not fully reflected on the validity of the results, reasoning that they were aware the screening was part of a research study.

#### 3.1.2 Effective DRS implementation in general practice in the future

##### 3.1.2.1 Ergonomic and spatial requirements

There was consensus on the critical importance of addressing spatial and ergonomic considerations in the implementation of DRS. A darkened screening room was proposed by many.

“*It requires a room, so that needs to be planned as well. It must be a room that's available for this purpose. And it doesn't have to be a big room, but it still needs to be darkened.”* (12)

Staff emphasised the importance of ensuring comfortable patient seating, recommending the use of both a height-adjustable table and chair to facilitate optimal ergonomic positioning.

“*We really have to have something that can be adjusted, both the table and the chair. You need to have something adjustable, so both bigger and smaller patients can sit comfortably. I actually think that's really important*.” (P6)

##### 3.1.2.2 Planning and coordination needs

Planning and coordination were emphasised, especially in practices with several staff involved, accessibility to the camera had to be ensured.

“*If you look at our schedule, all three of us conduct annual diabetes consultations at the same time, so the accessibility of the equipment needs to be ensured for us. We need the accessibility; it requires some level of planning.” (P12)*

Additionally, staff expressed a willingness to conduct the screenings if implemented, though a few suggested that medical students or social and health care workers could in the future conduct the screening as well.

“*The task itself is fine, and I'm not even sure it requires a nurse to perform it. I'm not certain—maybe a social and healthcare assistant could do it as well?” (P10)*

##### 3.1.2.3 Staff desire for more knowledge

Most staff indicated a desire for more knowledge about DR, key considerations during the screening process, and the ability to address patient questions in the context of broader implementation.

“*Of course, if this is something we are going to do, then we'll need a bit more background knowledge about it—that's how I feel… I might need to learn more about this eye disease, as this is not something I deal with in my everyday work.” (P21)*

Few staff felt confident using existing knowledge for the screening, noting that patients with DR alterations would be referred to an ophthalmologist for further information. Overall, there was consensus that DR was the area of diabetes care staffs were least familiar with.

##### 3.1.2.4 Screening in annual consultation

Most staff proposed integrating DRS into annual diabetes consultations as the most practical approach, highlighting the need for an additional 5–10 min to accommodate the procedure. Two staff members expressed uncertainty about including the screening in the annual consultation, citing concerns that it might overwhelm the patient. Nevertheless, both agreed that the screening should be incorporated into an existing patient visit.

##### 3.1.2.5 Financial conditions

Staff referenced the financial conditions as essential for implementing DRS in general practice. Staff questioned who would be responsible for funding the purchase of the camera and how practices would be compensated for conducting screenings.

“*There will always be financial considerations, such as who should purchase the equipment. If we are the ones to buy it, what compensation will we receive for using it?”* (P1)

Two staff members mentioned that financial compensation should directly benefit individuals performing the screenings, highlighting the importance of aligning salary with the increased competencies required for the task.

##### 3.1.2.6 Defined plan for ophthalmologist involvement

Staff explained the importance of knowing exactly when the ophthalmologist should be involved if implemented. Several staff members noted themselves that when DR alterations were detected, patients should be referred to an ophthalmologist for follow-up.

“*I imagine that if the camera indicates that there are some DR alterations, a referral to an ophthalmologist is needed, or I would need some kind of guidance on what to do next.” (P12)*

Two staff members highlighted the importance of ensuring that patients understood that the screening only covered DR and could not substitute a full ophthalmological examination.

#### 3.1.3 Trust and approval of AI-based screening in general practice

graphApproval of DRS in general practice Staff supported implementation of DRS in general practice. Staff described DRS as not fundamentally different in terms of time consumption from other tasks already performed in general practice.

“*The idea of having such equipment here, which might only take 5-6 minutes to use in total, isn't any different from what we do with throat swabs, ECGs, lung function tests, and CRP tests. So, I don't see any problem with that.” (P1)*

A few highlighted a limitation of implementing DRS in general practice: their inability to independently interpret the images, unlike other measurements performed in diabetes care.

“*The disadvantage is that we are not able to interpret the image; unlike with an ECG, where the machine provides its interpretation, but we always have our own as well. We wouldn't really be able to do that with a retinal image.” (P14)*

##### 3.1.3.1 Validity as the foundation for technological trust and implementation

The validity of the results was essential for the staffs' trust and accept of the screening into general practice.

“*If it is valid, and it works as intended, I have no doubt that it could easily be implemented here. There would be no problem with that.” (P3)*

A few staff members expressed that if the screening was implemented staff would have no choice but to trust the results.

##### 3.1.3.2 AI as a screening tool

Staffs were generally not concerned regarding their use of AI in this screening, with several pointing out that AI is already widely utilised in various aspects of daily life and is generally seen as an inevitable part of the future.

“*So, I'm not nervous about it being artificial intelligence. No, it's the future, that's for sure.” (P20)*

#### 3.1.4 Implications of DRS in general practice

##### 3.1.4.1 Increased accessibility and attendance

Staff believed that implementing DRS in general practice could increase patient accessibility and noted positive patient feedback on the screening. Furthermore, the established patient-provider relationship and the absence of eye drops were seen as factors that could further enhance patient convenience of DRS in general practice.

“*All patients expressed that they would like it to be part of their consultation here… I believe there are several factors involved, such as the convenience of simply coming here and the fact they meet someone they know well, which provides them with a sense of comfort. And, they don't need to have their eyes dilated, so it's easier for them to go home afterward—they can drive or cycle themselves.” (P17)*

Most staff highlighted that screening in general practice could increase DRS attendance by reaching patients who don't visit the ophthalmologist, potentially leading to earlier detection.

##### 3.1.4.2 Avoidance of ophthalmology visits

Screening in general practice was viewed by most as an opportunity to save patients' time, allowing those without detected alterations to avoid visiting an ophthalmologist for DR evaluation. A few staff members highlighted the advantage of patients not needing to take time off work.

“*They wouldn't have to make an appointment with the ophthalmologist and take time off work again—they could get everything done while they were already here. This way, they wouldn't need to take time off work twice.” (P19)*

Several staff members noted that general practice could sort healthy eyes, ensuring that ophthalmologists only manage patients with detected alterations. One staff member expressed ophthalmologists have long waiting lists while spending time examining many patients without DR alterations:

“*Ophthalmologists have long waiting list and could use their resources for other things. And when I look at the results, and there are no alterations, it means ophthalmologists are seeing many healthy eyes, which is silly.” (P18)*

##### 3.1.4.3 Increased responsibilities in general practice

While staff believed that DRS would be a manageable task, they emphasised the importance of being cautious taking on additional responsibilities without adequate resources. Most did not perceive the screening as an additional burden, but rather as a natural extension of the diverse roles that especially nurses already perform. However, it was emphasised that any new task, such as DRS, should provide value for both patients and practice without placing undue pressure on the workload:

“*Generally, it is my clear impression that we need to be very cautious about taking on additional tasks in general practice until we get more colleagues… It's important to ensure that the time invested provides the benefits, both for the patients and for ourselves, that correspond to the effort we put into it.” (P1)*

Some mentioned that DRS in general practice could enhance the quality of diabetes care by adding a new dimension to diabetes management. One individual expressed a desire to expand their provider role and take on additional tasks to contribute further to patient care:

“*I want to be able to do what we need to do and be at the forefront and do everything we can for the patients. That is the most important task, so the more we can do out here, the better, because we're the ones who see the patients, we're the ones who are in touch with them. So, it's definitely an advantage.” (P17)*

## 4 Discussion

### 4.1 Key findings

This study provided valuable insights into staff experiences and perspectives on conducting AI-assisted DRS in general practice. Key factors highlighted from implementing DRS in daily practice included logistical adaptations, challenges encountered during the process, and staff perceptions of the ease of performing DRS. For successful future implementation, staff emphasised the importance of the physical setup for screening, financial considerations, and a desire for increased knowledge about DRS. Staff expressed trust in and approval of the screening, though this was closely tied to the validity of the results. The implications of DRS in general practice, according to staff, could include increased convenience and attendance for patients, avoidance of ophthalmology visits for those without DR alterations, but would also include an increased responsibilities to general practice.

### 4.2 Findings in relation to previous studies

Since DRS is typically conducted in ophthalmology practices, this study, along with previous research (12), points that environmental factors do not always align optimally with the screening setup. Beede et al. (12) identified that DRS in some practices were conducted in shared or partially lit spaces, resulting in some screenings being performed under full illumination. This may negatively have impacted image quality but was most likely the available option within the clinical setting. Similarly, this study found staff using creative solutions to darken rooms sufficiently, and screenings were reported conducted in hallways or shared spaces due to practical constraints in the clinical setting. Therefore, careful evaluating practice's physical layout and workflow is crucial to minimise external factors that could negatively impact the screening process.

Staff did not exhibit significant resistance towards using AI; however, they emphasised that their trust in AI was dependent on technological validity. Similarly, previous studies have highlighted that health care staff value evidence supporting the validity and reliability of AI tools as essential for enhancing their acceptance and trust in such technologies (5). Acceptance of a new technology is according to Technology Acceptance Model (TAM) dependent on individuals' perceived usefulness and ease of use (26). Ease of use has also been predicted as important for successful implementation of DRS into primary care (5, 15) and staff in this study described the screening process was quick and easy to use. However, previous studies have reported less convincing results regarding ease of use when employing different screening equipment and AI software (5). Selection of fundus camera and analysis software should therefore be carefully considered to ensure that users experience ease of use. The fundus camera selected in this study was user-friendly and featured multiple automated functions, complemented by automated analysis. According to TAM, the likelihood of accepting a technology increase when users perceive it as enhancing their performance while requiring minimal effort to use (26). In this study, enhanced performance for staff can be described as their improved competency in diabetes management. Staff noted that DRS could improve quality of diabetes care, which they regarded as a clear advantage. From TAM perspective, these factors likely support staff acceptance of the technology.

Adequate training is highlighted as a critical factor for successful implementation of AI tools (15, 16, 27), a finding supported by this study. AI tools enable task shifting to less specialised personnel by performing tasks traditionally carried out by specialists (28) in this study, analysis of fundus images. However, insufficient training can pose challenges, as a lack of knowledge about advanced technologies often leads to practical difficulties and hesitancy in adoption (15, 27). Prior research emphasises that targeted education and training not only enhance the experience for healthcare professionals but also facilitate the adoption of digital health technologies, whereas a lack of training is associated with negative experiences and reduced utilisation (27). It would therefore be advantageous to implement training that encompasses both the clinical and technical aspects of DRS.

Primary care is viewed as an ideal setting for the implementation of various AI solutions, given their role as the first point of care (1, 2). This is particularly advantageous because AI tools can enhance efficiency and support clinical decision-making (1). However, general practices are already under significant pressure due to heavy workloads, and general practitioners have some concerns regarding whether AI solutions will alleviate or exacerbate this burden (29). While certain AI tools have the potential to assist or take over tasks (1), the introduction of new responsibilities, such as DRS, inevitably adds to the workload. As highlighted by staff in this study, it is essential that any new task implemented in general practice is meaningful both to the practice and to patients. Staff did not view the screening as an additional burden but describe it as an opportunity to improve the quality of diabetes care. Research on patients perception on AI-assisted DR screenings in primary care has generally been positive, with many expressing satisfaction and a willingness to use this method again (4, 17, 30). Nevertheless, some patients remain hesitant about AI replacing ophthalmologists in diagnostic roles (30, 31). Therefore, patients' perceptions towards AI-assisted DRS in primary care should be thoroughly considered and further explored when planning its implementation.

Aligning with findings from prior studies (13–15), this study emphasises the critical role of economic considerations. A Singaporean study identified a combined AI-human approach—where AI analysed images and humans graded positive cases—as the most cost-effective (32). Similarly, a Scottish study reported a 46.7% cost reduction by replacing first-level human assessment with AI (33), while a UK study observed savings of 12.8–21.0% (34). These differences likely stem from variations in screening program design, classification criteria, workforce costs, and pricing models (33, 34), emphasising the importance of conducting cost-effectiveness analyses in the targeted country.

The AI software used in this study employs SVML to classify the presence or absence of DR. In contrast, newer AI software based on deep learning can classify DR according to the International Clinical Diabetic Retinopathy (ICDR) severity scale; however, its “black-box” nature limits explainability, as it relies on pattern recognition rather than traditional lesion identification (28, 35). A limitation of the software used in this study is its inability to grade DR according to the ICDR scale. However, it offers high explainability by providing black outlines around the detected red lesions, facilitating the identification of pathological changes. The software has been described as a useful screening tool for distinguishing between eyes with and without DR (36), which may be sufficient for DRS in general practice, where patients with retinal changes need to be referred to an ophthalmologist. The necessity of ICDR-based grading should be carefully considered depending on the setting and intended use.

### 4.3 Strengths and limitations

A strength of this study is that staff gained hands-on experience with screening within a real clinical setting, identifying practical advantages and areas for improvement, which provided valuable insights for future DRS implementation.

This study has methodological limitations. This study could have been strengthened by applying a theoretical framework, such as the Consolidated Framework for Implementation Research (37) or Normalisation Process Theory (38). While we did not apply a theoretical framework in this feasibility study, as our primary aim was to explore initial feasibility through staff experiences and perspectives, future research should consider integrating relevant theoretical frameworks to strengthen the understanding of implementation processes and support long-term adoption.

The interviewers had limited experience with qualitative research, which may have influenced both the interview process and data analysis. To enhance trustworthiness and credibility of the data analysis, two researchers were involved. The sample size of interview participants was not determined through an analytical assessment of data saturation, but was instead defined pragmatically. We included staff from all participating practices, as different workflows, staff availability, and organisational structures could have influenced their experiences with DRS. These factors, we believe, were essential to explore to gain a comprehensive understanding of staff experiences.

The potential for selection bias is a limitation of this study, as participating practices had to be willing to adapt their workflows to incorporate screening. This may have attracted practices with a greater interest in AI-assisted DRS. Furthermore, the responsibility for T2D care in Danish general practices often lies with nurses under the supervision of general practitioners. The purpose of this feasibility study was not to select specific staff based on their profession, but rather to gain insight into the experiences and perspectives of those conducting the screening. Staff conducting DRS staff were selected by the practices based on their involvement in T2D care, however, the overrepresentation of nurses may limit the generalisability of the results to clinics with a different workflow.

### 4.4 Implications for policymakers

If DRS in general practice is a policy objective, a fundamental restructuring of the current DRS process is necessary. It requires internal adjustments within practices and given the variation in physical layouts and workflows, it is essential to provide practices with autonomy in decision-making regarding how to implement DRS into each practice. Additionally, it is essential to consider external factors such as who will bear the cost of the equipment and how general practices will be compensated conducting DRS. Moreover, clinical guidelines must include recommendations for handling DRS and when ophthalmologist referral is necessary.

### 4.5 Future research

Integrating new technologies into healthcare is inherently complex. This study first underscores the need to address both practical and systemic factors for successful implementation of DRS in general practice. To support this process, future research should investigate key elements to further inform a Health Technology Assessment (39), such as patient perspectives, an economic evaluation of the cost-effectiveness of adopting AI-assisted DRS in Danish healthcare and further assess the validity of DRS in real-world clinical settings.

Second, the AI software utilised in this study has primarily been tested on a Caucasian population (36), and its generalisability to other ethnic groups remains uncertain. This limitation is also observed in other retinal AI software, which has frequently been tested on specific ethnic populations (28, 40). Furthermore, studies have pinpointed that the image quality and accuracy of AI models can vary depending on the camera used (40). Software requirements and camera models are critical factors to consider for optimal implementation, warranting further investigation.

## 5 Conclusion

Through staff interviews, we gained insights into the experiences and perspectives on conducting DRS in general practice. Integrating DRS required logistical adaptations, and while staff generally described the screening as quick and easy, some encountered challenges that required problem-solving. Key factors for successful future implementation included the clinical setup, financial considerations, a defined plan for ophthalmologist involvement and the need for greater knowledge about DRS for staff. Staff expressed trust in and approval of DRS, though this was contingent on the validity of screening results. The introduction of DRS in general practice could enhance patient convenience and attendance, reduce unnecessary ophthalmology referrals, but also increase responsibilities for general practice. This study provides a foundation for future research on optimising AI-assisted DRS implementation in general practice, while recognising that the insights gained may also inform broader primary care context.

## Data Availability

The datasets presented in this article are not readily available because of participant privacy concerns. Requests to access the datasets should be directed to Malene Krogh at Malenekrogh@dcm.aau.dk.
